# A two-step actin polymerization mechanism drives dendrite branching

**DOI:** 10.1186/s13064-021-00154-0

**Published:** 2021-07-19

**Authors:** Rebecca Shi, Daniel A. Kramer, Baoyu Chen, Kang Shen

**Affiliations:** 1 Department of Biology, Stanford University, Stanford, CA 94305 USA; 2 Neurosciences IDP, Stanford University, Stanford, CA 94305 USA; 3 Roy J. Carver Department of Biochemistry, Biophysics and Molecular Biology, Iowa State University, Ames, IA 50011 USA; 4 Howard Hughes Medical Institute, Stanford University, Stanford, CA 94305 USA

**Keywords:** Dendrite branching, Dendrite morphogenesis, Actin polymerization, Ena/VASP, WAVE regulatory complex

## Abstract

**Background:**

Dendrite morphogenesis plays an essential role in establishing the connectivity and receptive fields of neurons during the development of the nervous system. To generate the diverse morphologies of branched dendrites, neurons use external cues and cell surface receptors to coordinate intracellular cytoskeletal organization; however, the molecular mechanisms of how this signaling forms branched dendrites are not fully understood.

**Methods:**

We performed in vivo time-lapse imaging of the PVD neuron in *C. elegans* in several mutants of actin regulatory proteins, such as the WAVE Regulatory Complex (WRC) and UNC-34 (homolog of Enabled/Vasodilator-stimulated phosphoprotein (Ena/VASP)). We examined the direct interaction between the WRC and UNC-34 and analyzed the localization of UNC-34 in vivo using transgenic worms expressing UNC-34 fused to GFP.

**Results:**

We identify a stereotyped sequence of morphological events during dendrite outgrowth in the PVD neuron in *C. elegans*. Specifically, local increases in width (“swellings”) give rise to filopodia to facilitate a “rapid growth and pause” mode of growth. In *unc-34* mutants, filopodia fail to form but swellings are intact. In WRC mutants, dendrite growth is largely absent, resulting from a lack of both swelling and filopodia formation. We also found that UNC-34 can directly bind to the WRC. Disrupting this binding by deleting the UNC-34 EVH1 domain prevented UNC-34 from localizing to swellings and dendrite tips, resulting in a stunted dendritic arbor and reduced filopodia outgrowth.

**Conclusions:**

We propose that regulators of branched and linear F-actin cooperate to establish dendritic branches. By combining our work with existing literature, we propose that the dendrite guidance receptor DMA-1 recruits the WRC, which polymerizes branched F-actin to generate “swellings” on a mother dendrite. Then, WRC recruits the actin elongation factor UNC-34/Ena/VASP to initiate growth of a new dendritic branch from the swelling, with the help of the actin-binding protein UNC-115/abLIM. Extension of existing dendrites also proceeds via swelling formation at the dendrite tip followed by UNC-34-mediated outgrowth. Following dendrite initiation and extension, the stabilization of branches by guidance receptors further recruits WRC, resulting in an iterative process to build a complex dendritic arbor.

**Supplementary Information:**

The online version contains supplementary material available at 10.1186/s13064-021-00154-0.

## Background

Neurite outgrowth and arborization are essential for the establishment and function of neural circuits. In particular, the morphology of dendrites is key for neuronal signal transmission and integration. Dendrite morphogenesis is often guided by extracellular cues recognized through a number of cell surface receptors, such as Semaphorins, DSCAMs, and protocadherins, which in turn instruct activities of various intracellular molecules leading to cytoskeletal reorganization [1, 2]. Similarly, activity-dependent dendritic arbor development also requires cytoskeletal signaling, including through Rho GTPase activity [3] or microtubule stabilization [4].

One major signaling axis for neurite outgrowth is the activation and membrane recruitment of the actin nucleation promotion factor, the WAVE Regulatory Complex (WRC), a pentameric complex comprising Sra1/Cyfip1, Nap1/Hem-2, Abi, HSPC300, and WAVE/Scar [5]. After being activated and recruited by diverse membrane receptors and the Rho family GTPase Rac1, the WRC can then stimulate the Arp2/3 complex to produce branched actin networks [6]. In addition, a distinct actin elongation factor, Ena/VASP, can be recruited downstream of the Netrin receptor, DCC, [7–9] to promote polarized filopodia formation during axon guidance. Ena/VASP can also be recruited by the Slit receptor, Robo, to mediate axon repulsion [10, 11]. Interestingly, these distinct actin regulators can act synergistically in several morphological processes. For example, the direct interaction between the WRC and Ena/VASP was shown to play an important role in photoreceptor axon targeting, oogenesis, and macrophage migration in *Drosophila*, lamellipodia formation during ventral closure of *C. elegans* embryogenesis, and collective axon extension in mice [12–14]. It is, however, largely unknown how the activity of distinct actin regulators is coordinated in complex morphological processes.

One hypothesis is that complex processes like neuronal morphogenesis integrate structurally distinct actin networks built by different actin regulators. For example, in dendritic spines of hippocampal pyramidal neurons, patches of loosely bundled linear actin filaments constitute the dendritic spine neck, whereas branched actin networks drive the expansion of spine heads [15]. Furthermore, Abl tyrosine kinase has been shown to downregulate Ena activity while in parallel activating Rac/WAVE signaling, thus coordinating the balance of linear actin bundles and branched actin networks to regulate *Drosophila* axon patterning [16]. Finally, branched actin networks generated via the Arp2/3 complex were found to provide sites for initiating new dendrite branches in *Drosophila* larval sensory da neurons [17]. Therefore, it is likely neurons control morphological changes by dynamically orchestrating the formation of distinct types of actin networks in a coherent manner.

In this work, we provide evidence in support of this hypothesis showing that in the *C. elegans* PVD neuron, the development of dendritic arbors requires two distinct, but cooperative, steps of actin assembly that involve the WRC, UNC-34 (Ena/VASP), and the actin-binding protein UNC-115 (abLIM). As a recently developed model system for studying dendritic morphogenesis, the PVD neuron elaborates complex dendritic arbors by extending a primary dendrite along the length of the worm, followed by orthogonal secondary, tertiary, and quaternary dendrites, which together create stereotyped “menorah”-like structures [18]. A multipartite ligand-receptor complex, consisting of the extracellular ligands SAX-7, MNR-1, and LECT-2, along with the PVD guidance receptor DMA-1 and its partner HPO-30, directs the growth of PVD dendrites along the epidermis and body wall muscles [19–23]. This ligand-receptor complex recruits actin regulators, including the Rac GEF TIAM-1 and the WRC, through the cytosolic domains of DMA-1 and HPO-30, respectively, which in turn produce F-actin in growing dendrite tips to drive dendrite branching [23–25]. However, the mechanisms of F-actin recruitment during distinct morphological steps during dendrite development, such as initiation of new branches and branch elongation, remain unclear.

Here, we show that new dendrite branch points are established first as local “swellings” along dendrites, from which filopodia sprout to enable rapid and efficient outgrowth of dendritic branches. The formation of swellings is mediated by the WRC, while the subsequent extension of filopodial branches is mediated by UNC-34/Ena/VASP and UNC-115/abLIM. Our data suggest that a direct interaction between the WRC and UNC-34 provides the mechanism by which two distinct actin assembling processes are coupled to drive dendrite arborization.

## Results

### Morphological characterization of “swellings” and filopodia during PVD dendrite outgrowth

**Fig. 1:**
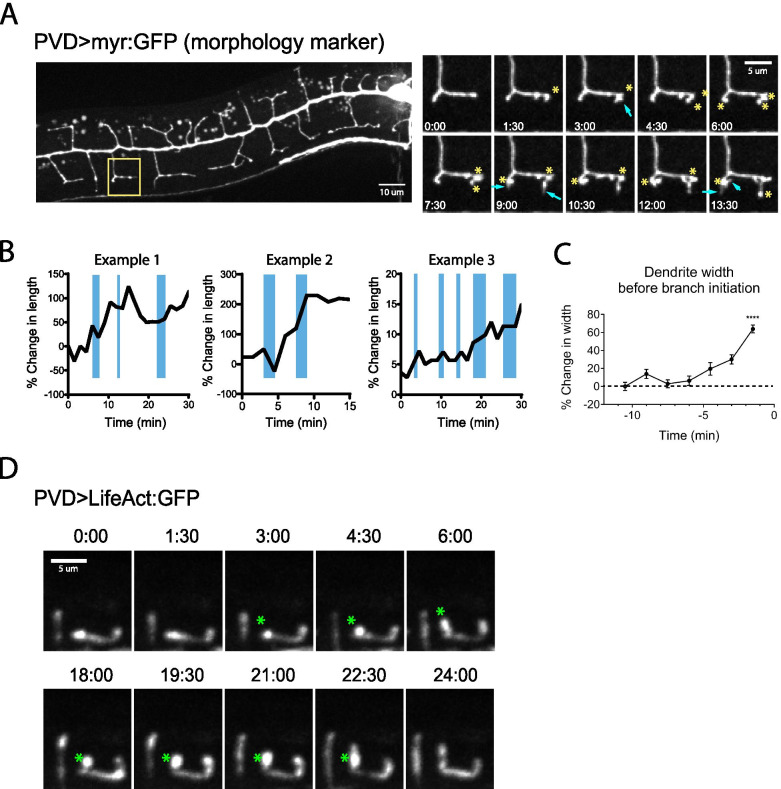
PVD dendrites exhibit local increases in width, or “swellings”, which precede filopodia-like outgrowth. **A** Live animal imaging showing a representative developing PVD dendrite in an L3 stage wild-type worm. PVD morphology is visualized by a myristoylated GFP marker unless indicated otherwise (ser2prom3 > myr-GFP). Yellow rectangle indicates the location of the magnified time-lapse shown on the right. Yellow asterisks indicate swellings; cyan arrows indicate filopodia emerging from swellings. **B** Three representative examples showing growth of an outgrowing quaternary branch. The presence of a swelling at the tip of the dendrite is denoted with blue bars. Note swellings that occur before rapid increases in length (filopodia). **C** Quantification of dendrite width at branching sites prior to branch initiation. Statistical comparison was performed between the -10.5 and -1.5 min timepoints using a two-tailed Mann–Whitney test (****, *p* < 0.0001, *n* = 14 dendrites). **D** Live animal imaging using a LifeAct:GFP marker expressed in PVD. Asterisks indicate swelling structures that show enrichment of F-actin before branch growth

To identify distinct morphological events during the development of PVD dendritic arbors, we performed in vivo live imaging of the PVD dendrite at the third larval stage (L3) of wild-type worms. At this stage, the dendrite elaborates its tertiary and quaternary branches (see Additional files 1 and 4). Using a myristoylated-GFP (myr-GFP) marker expressed specifically in PVD, we observed many dynamic growth and retraction events that ultimately led to the formation of menorah-like branches, consistent with previous findings [18, 26]. In addition, we noticed that the growth of the quaternary branches often occurred through a series of rapid filopodia-like growth events, which were interspersed by several pauses (Fig. 1A-C). When we plotted the growth of quaternary dendrites over time (Fig. 1B, three independent examples) and examined dendrite morphology during growth events, we found that existing dendrites often exhibited a local increase in width (blue bars) right before the initiation of new branches or further extension of existing dendrites. Quantification of dendrite width showed a significant increase 1–2 min before new branch initiation (Fig. 1C), and filopodia emerged rapidly from these enlarged locations (Fig. 1B-C). We herein refer to these local increases in width as “swellings”. Using a LifeAct:GFP marker, we confirmed that F-actin is enriched in these swellings prior to initiation or elongation of the dendritic branches (Fig. 1D). These data suggest that the swellings are actin-based, which is consistent with previous reports that PVD dendrite growth is primarily driven by actin polymerization [23, 24]. Finally, we noticed that filopodia grew from the swellings in various directions, but only a subset of the filopodia were stabilized as quaternary dendrites (see Additional files 1 and 4). This behavior is consistent with the notion that filopodia are used to probe extracellular environments, perhaps in search of external guidance cues such as the patterned SAX-7 stripes along which PVD grows [27].

### Filopodia formation during PVD dendrite outgrowth depends on unc-34 (Ena/VASP) and unc-115 (abLIM)

**Fig. 2:**
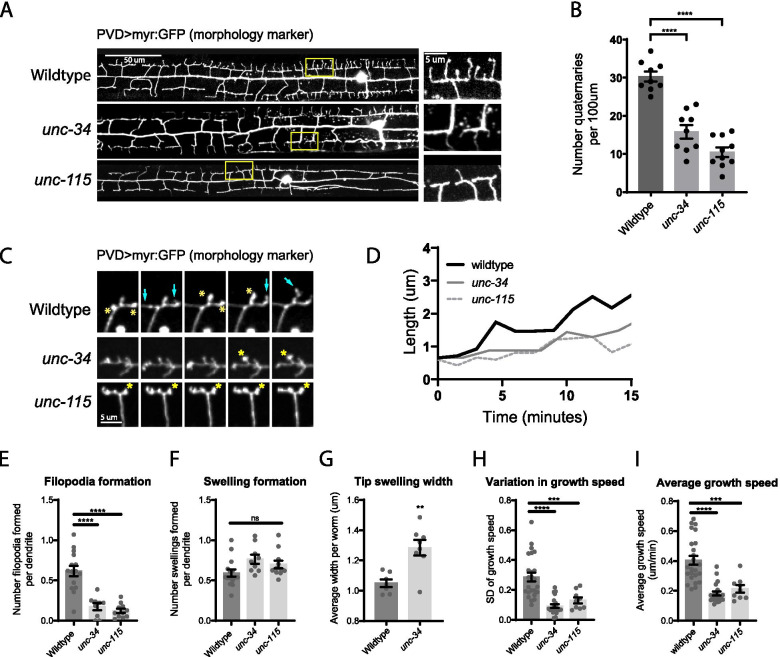
Filopodial outgrowths depend on UNC-34 (Ena/VASP) and UNC-115 (abLIM). **A** Representative images of PVD dendrite morphology in wild-type, *unc-34(null)*, and *unc-115(null)* animals at the L4 stage. Yellow rectangles indicate locations of magnified views on the right. **B** Quantification of number of quaternary branches in a region 100 µm anterior to the PVD cell body. Statistical comparison was performed using Brown-Forsythe ANOVA with Dunnett’s multiple comparisons test (****, *p* < 0.0001; *n* = 9 worms for wild-type and *unc-34*; *n* = 10 worms for *unc-115*). **C** Live animal imaging of developing PVD dendrites in wild type, *unc-34* mutant, and *unc-115* mutant. Cyan arrows indicate filopodia that emerge in wild type. Yellow asterisks indicate swellings, which fail to initiate filopodia in *unc-34* and *unc-115* mutants. Scale bar is 5 µm. **D** Representative traces showing growth of individual dendrites of indicated genotypes. **E** and **F** Quantification of filopodia (**E**) and swelling formation (**F**). Statistical comparison was performed using Brown-Forsythe ANOVA with Dunnett’s multiple comparisons test (****, *p* < 0.0001; ns, *p* > 0.05; *n* = 15 worms for wild-type; *n* = 9 worms for *unc-34*; *n* = 11 worms for *unc-115*). **G** Quantification of tip swelling width. Each data point is the averaged width of swellings measured from 5 to 10 dendrites within a single worm. Statistical comparison was performed with a two-tailed Mann–Whitney test (**, *p* < 0.01; *n* = 7 worms for wild-type; *n* = 8 worms for *unc-34*). **H** and **I** Quantification of standard deviation (SD) (**H**) and average (**I**) of dendrite branch growth speed. Lower SD indicates more consistent, gradual increases in length. See Methods for details. Statistical comparison was performed using Brown-Forsythe ANOVA with Dunnett’s multiple comparisons test (****, *p* < 0.0001; ***, *p* < 0.001; *n* = 24 branches for wild-type; *n* = 21 branches for *unc-34*; *n* = 9 branches for *unc-115*)

To identify the molecular mechanisms underlying the formation of the actin-rich swellings and filopodia observed above, we next examined PVD outgrowth in mutants of various actin regulators. We first focused on *unc-34*, the sole *C. elegans* Ena/VASP protein homolog, and *unc-115,* an actin-binding LIM protein (abLIM) previously implicated in neurite outgrowth. Ena/VASP proteins play a critical role in polymerizing linear F-actin to initiate and extend filopodia [28]. UNC-115 is thought to be a Rac effector that can act downstream of Netrin [9, 29] and has been shown to regulate axon guidance by affecting the dynamics of filopodia and lamellipodia in axonal growth cones [9, 30, 31]. Specifically, overexpression of membrane-targeted UNC-115 caused the formation of ectopic neurites, lamellipodia, and filopodia in the *C. elegans* PDE neuron, and deletion of the actin-binding VHD domain greatly reduced these effects [30]. We found that null mutants of *unc-34* and null mutants of *unc-115* both exhibited a strong loss of quaternary branches (Fig. 2A-B), suggesting that these actin regulators are also required for reorganizing the actin cytoskeleton during PVD dendrite morphogenesis.

We next used live animal imaging to examine if the *unc-34* or *unc-115* mutants affected swelling or filopodia formation during PVD dendrite outgrowth (Fig. 2C-I; Additional files 2 and 3). We found that in both *unc-34* and *unc-115* mutants, filopodia formation was drastically reduced, whereas dendrite swellings remained unaffected (Fig. 2E–F). Furthermore, lack of filopodia in *unc-34* or *unc-115* mutants switched the wild-type “rapid-growth-with-pauses” mode of dendrite extension to a slow and steady outgrowth mode characterized by reduced speed of remaining outgrowth events (Fig. 2D, I, Fig. S1), and less variability in the speed of such outgrowth (Fig. 2H). In particular, *unc-34* mutants exhibited large, persistent growth cones at the tip of growing dendrites, which occasionally split to create new dendritic branches (Fig. 2C, Fig. S1, Additional file 2). These growth cones were wider than wild type growth cones (Fig. 2G) and extended steadily forward (Fig. 2H), reminiscent of the increased persistence of lamellipodia observed in migrating fibroblasts depleted of Ena/VASP proteins [32]. Taken together, our results suggest that both UNC-34 and UNC-115 play a key role in filopodia formation during PVD dendrite branching. The fast-growing filopodia ensure efficient growth of dendritic arbors, especially in the initiation of new quaternary branches. Because *unc-34* and *unc-115* mutants have also been reported to disrupt filopodia initiation in axonal growth cones [33], similar cytoskeletal mechanisms may also be involved in axon development.

### Swelling formation during PVD dendrite outgrowth depends on the WAVE Regulatory Complex

**Fig. 3:**
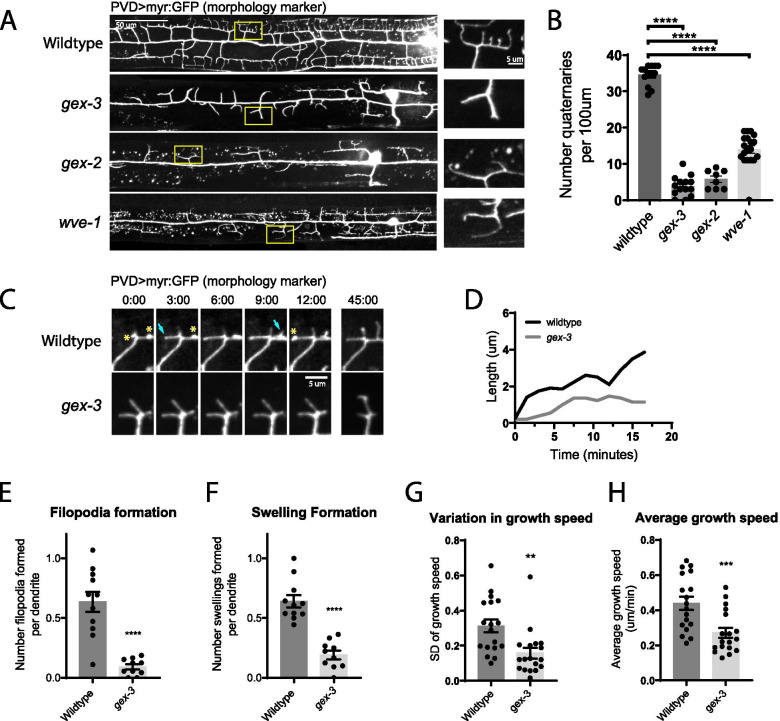
WRC mutants lack both filopodia and swellings. **A** Representative images of PVD dendrite morphology in wild-type, *gex-3*, *gex-2*, and *wve-1* null animals at the L4 stage. Yellow rectangles indicate locations of magnified views on the right. **B** Quantification of number of quaternary branches in a region 100 µm anterior to the PVD cell body. Statistical comparison was performed using Brown-Forsythe ANOVA with Dunnett’s multiple comparisons test (****, *p* < 0.0001; *n* = 13 worms for wild-type; *n* = 14 worms for *gex-3*; *n* = 8 worms for *gex-2*, and *n* = 19 worms for *wve-1*). **C** Live animal imaging of developing PVD dendrites in L3 stage animals in wild-type and *gex-*3 animals. Yellow asterisks indicate swelling events that gave rise to filopodial growths (cyan arrows), which are largely absent in *gex-3* worms. Note the overall lack of growth in *gex-3* mutants compared to wild type. Scale bar is 5 µm. **D** Representative traces showing growth of individual dendrites of indicated genotypes. **E** and **F** Quantification of filopodia and swelling formation as described in Fig. 2. Statistical comparison was performed using two-tailed Mann–Whitney test (****, *p* < 0.0001; *n* = 10 worms for wild-type; *n* = 11 worms for *gex-3*). **G** and **H** Quantification of positive growth events in indicated genotypes as described in Fig. 2. Statistical comparison was performed using two-tailed Mann–Whitney test (**, *p* < 0.01; ***, *p* < 0.001; *n* = 24 branches for wild-type; *n* = 18 branches for *gex-3*)

Because neither *unc-34* nor *unc-115* mutants reduced swellings, we next asked what molecular mechanisms are required to form these structures. We previously showed that the WAVE Regulatory Complex (WRC) plays a critical role in PVD dendrite development through its direct interaction with the guidance receptor complex that mediates PVD outgrowth [23]. Consistent with our previous results, we found that mutants in the WRC components Sra1, Nap1/Hem-2, or WAVE/Scar (named *gex-2, gex-3*, and *wve-1* in *C. elegans*, respectively) all showed severely truncated dendritic arbors at the L4 stage (Fig. 3A-B). All three WRC mutants exhibited a maternal effect lethal phenotype, where the offspring of heterozygous mothers are viable but sterile, suggesting that the alleles are indeed strong loss-of-function [34]. However, because the maternal WRC is clearly sufficient for the animals to develop to adulthood, it is possible that variation in the amount of residual WRC activity may account for slight differences in phenotype across the three mutants, such as the slightly weaker phenotype of the *wve-1* mutant (Fig. 3B).

To test if the WRC is required to form swellings, we performed live animal imaging on *gex-3* mutants and found that tertiary dendrites failed to form both swellings and filopodia (Fig. 3E–F; Additional file 5). As a result, we observed very few growth events overall (Fig. 3G-H; compare Additional files 4 and 5). We note that the *gex-3* phenotype is distinct from the phenotypes of *unc-34* and *unc-115* mutants in that both swellings and filopodia were absent in *gex-3* mutants, whereas only filopodia, but not swellings, were disrupted in *unc-34* and *unc-115* mutants. Since filopodia arise from swellings in wild-type animals, it is plausible that the WRC promotes the formation of swellings, which then determines the locations for filopodia growth driven by UNC-34 and UNC-115. A similar mechanism, termed the “convergent elongation” model, has previously been proposed to describe filopodia formation from branched actin networks [35]. In this model, actin filaments from a branched network that is bundled together to form nascent filopodia. This mechanism has been proposed to explain filopodia formation in ex vivo chick sensory axons and cultured primary neuron growth cones [36, 37].

### The WAVE Regulatory Complex can directly bind to UNC-34 through PPR-EVH1 interactions

What mechanisms might coordinate the local WRC activity in swellings with the UNC-34 or UNC-115 activity in filopodia formation? One possibility is that the WRC recruits Ena/VASP proteins through a direct interaction between the poly-proline region (PPR) of Abi and WVE, and the EVH1 domain of Ena/VASP. Such an interaction was previously observed between human and *Drosophila* proteins, but the exact PPR sequences in Abi and WVE are divergent between various animal species [12].

**Fig. 4:**
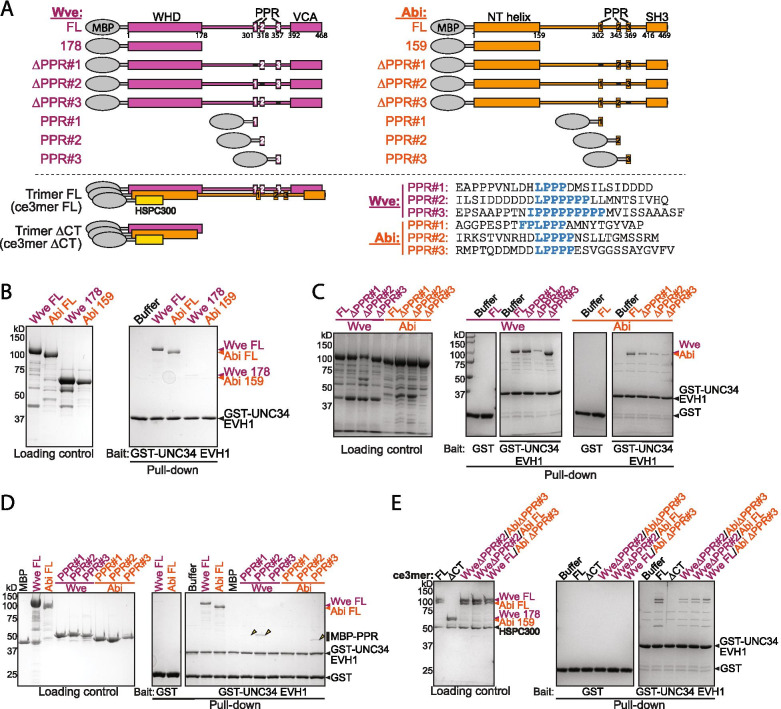
UNC-34 directly binds to PPRs of WVE and Abi. **A** Cartoon representation of the WVE, Abi, and trimer constructs used in binding assays. PPR sequences predicted to bind to EVH1 domain are shown in the bottom right, with the binding motif in blue text. In ∆PPR constructs, the blue-colored sequences are replaced with the same number of amino acids containing “AGGGG”. **B**-**E** Coomassie-blue stained SDS PAGE gels showing GST-UNC-34 EVH1 pulling down purified WVE and Abi proteins. Images compare full-length or truncated proteins lacking the PPR sequences (**B**); full-length WVE and Abi carrying mutations that disrupt indicated PPR sequences (**C**); isolated PPR sequences (**D**); and various WVE/Abi/HSPC300 trimeric subcomplexes (**E**). Gel images are representative of at least two independent repeats

To verify whether the UNC-34 EVH1 domain also directly binds to the *C. elegans* WRC (ceWRC), we recombinantly purified MBP (Maltose binding protein)-tagged full-length (FL) Abi and WVE, MBP-tagged fragments of Abi and WVE, and a GST-tagged EVH1 domain of UNC-34 for pull-down assays (Fig. 4A). Consistent with previous reports [12, 28], GST-UNC34 EVH1 retained both Abi FL and WVE FL (Fig. 4B), but not truncated proteins that lack the PPR sequences (WVE 178 and Abi 159, Fig. 4B). By mutating each of the three PPR sequences in WVE and Abi, which we predicted to bind to EVH1 using the “LPPPP” motif [12], we determined that multiple PPR sequences in both WVE and Abi contributed to the binding to EVH1, albeit at different levels. Particularly, deletion of WVE PPR#2, Abi PPR#3, and to a lesser extent Abi PPR#1 or PPR#2, clearly decreased the binding of UNC-34 EVH1 (WVE ΔPPR#2, Abi ΔPPR#1, 2 & 3; Fig. 4A, C). Note that although deleting WVE PPR#1 or #3 did not appear to reduce the binding, deletion of WVE PPR#2 still showed a residual, but clear interaction, suggesting PPR#1 and #3 remaining in the full-length WVE had a weak, but specific contribution to the binding. This result was further confirmed by direct binding between GST-UNC-34 EVH1 and isolated fragments containing individual WVE PPR#2 or Abi PPR#3 (Fig. 4A, D). In addition, we observed weak, but specific interaction of the isolated WVE PPR#1 (Fig. 4D). We could not detect binding of the isolated WVE PPR#3, Abi PPR#1 or #2, likely due to the limit of pull-down assays in detecting weak interactions of affinity in, empirically, tens of micromolar range (Fig. 4D). The slight discrepancy between full-length proteins, which contain multiple PPRs, and isolated PPRs likely reflects an avidity effect of multivalent interactions in sustaining a binding through otherwise weak individual ones.

Finally, we tested if UNC-34 EVH1 bound to the trimeric subcomplex of the WRC containing Abi, WVE, and HSPC300 (ce3mer; Fig. 4A). We used the trimeric subcomplex as a surrogate for the whole ceWRC pentamer because the subcomplex contained all PPRs found in the ceWRC pentamer, behaved well in biochemical reconstitution, and was less difficult to purify than the pentameric complex [23]. We found that in the context of the ceWRC subcomplex, entirely removing the PPR regions from both WVE and Abi abolished the binding to UNC34 EVH1, whereas mutating either WVE PPR#2, or Abi PPR#3, or both, partially reduced binding (Fig. 4E). This result further supports the notion that multiple WVE and Abi PPR sequences interact with UNC-34 EVH1. Although individual PPRs have different, sometimes weak, affinity (Fig. 4B-C), the availability of many PPRs in the trimer provides avidity (and possibly some cooperativity) to sustain a robust binding to EVH1, which could overcome deletion of the two PPRs with strongest affinity (Fig. 4E). Taken together, we conclude that the ceWRC can directly bind to UNC-34 through multiple PPR-EVH1 interactions.

### The EVH1 domain of UNC-34 is necessary for UNC-34 localization and function in vivo

**Fig. 5:**
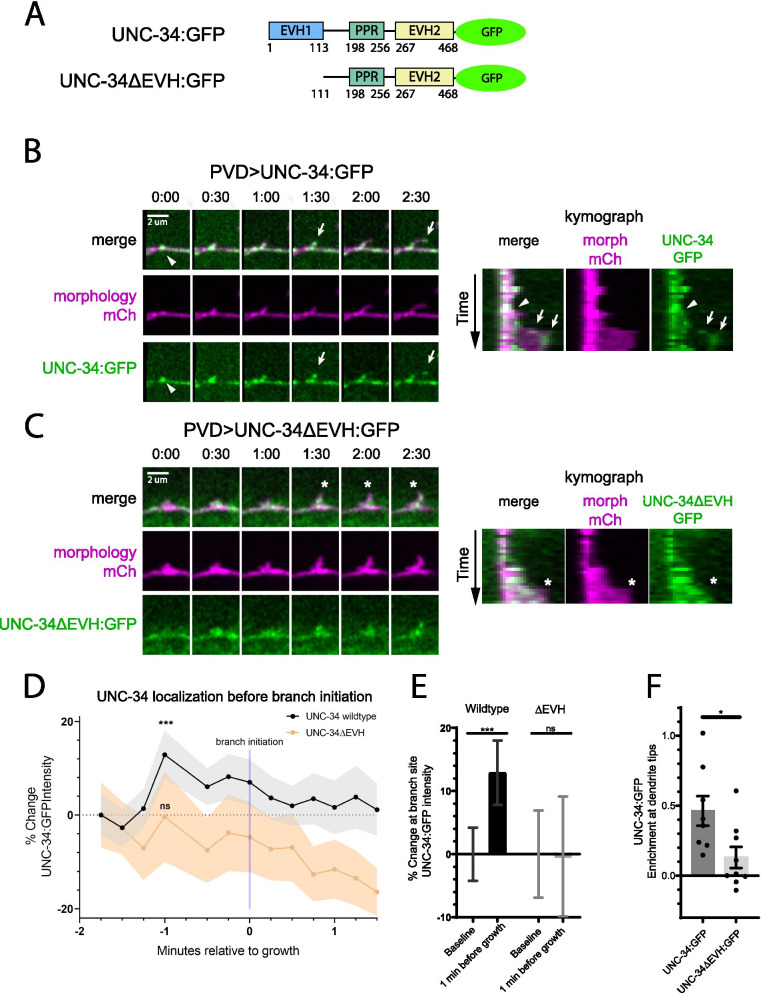
UNC-34 EVH1 domain is required for UNC-34 localization. **A** Schematic showing UNC-34 constructs used in the rescue experiments. **B** and **C** Live animal imaging of developing PVD dendrites in L3 stage wild-type animals that express wild-type UNC-34:GFP (**B**), or *unc-34* null animals that express UNC-34ΔEVH:GFP (**C**). PVD dendrite morphology was visualized using a myristoylated-mCherry morphology marker (ser2prom3 > myr-mCh). White arrows indicate tip localization of UNC-34:GFP in filopodia. White arrowheads indicate swelling localization of UNC-34:GFP before filopodia initiation. White asterisk indicates large growth cone-mediated growth lacking UNC-34ΔEVH:GFP enrichment at tip. Scale bar is 2 µm. Kymographs of dendrite growth are presented on the right. **D** and **E** Quantification of UNC-34:GFP enrichment to swellings before branch initiation. GFP intensity was measured at branching sites prior to branch outgrowth. Note the peak of UNC-34:GFP signal at 1 min before branch formation is absent in worms expressing the ΔEVH construct. Statistical comparison was performed between the -1.75 min (baseline) and -1.0 min timepoints using the Wilcoxon matched-pairs signed rank test. (***, *p* < 0.001; ns, *p* > 0.05; *n* = 28 branches for wild-type UNC-34:GFP; *n* = 25 branches for UNC-34ΔEVH:GFP). **F** Quantification of UNC-34:GFP enrichment at dendrite tips. GFP intensity was measured at the tip of a growing dendrite and normalized as % increase in intensity compared to GFP intensity along the dendrite shaft. Each data point is the average measured from 4–10 branches from a single worm. Statistical comparison was performed using the two-tailed Mann–Whitney test (*, *p* < 0.05; *n* = 8 worms for wild-type, *n* = 9 worms for UNC-34ΔEVH:GFP)

**Fig. 6:**
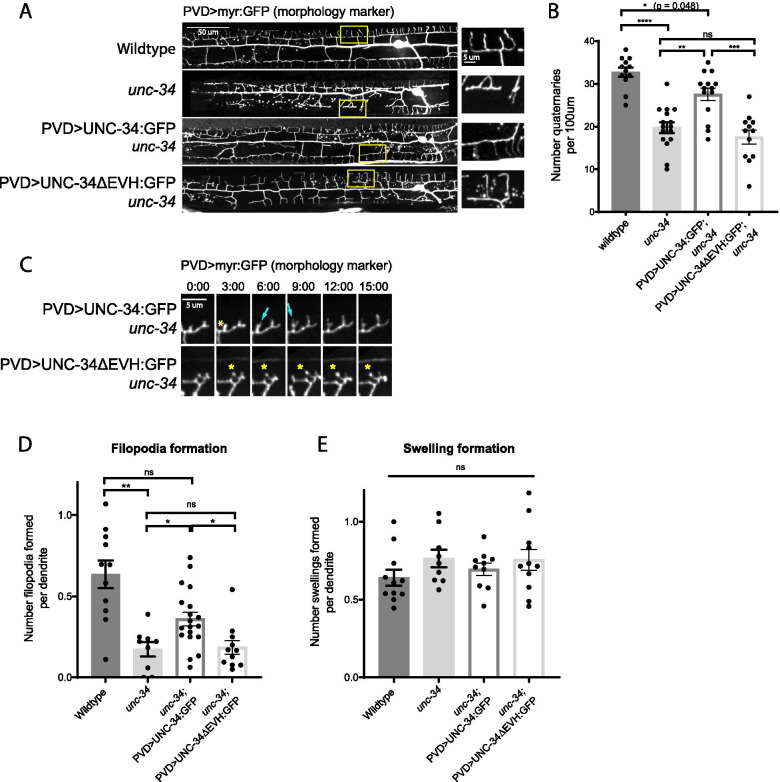
UNC-34 EVH1 domain is required for filopodia initiation and dendrite branch formation. **A** Representative images of PVD dendrite morphology in wild-type, *unc-34 null*, UNC-34 wild-type rescue, and UNC-34ΔEVH rescue animals at the L4 stage. Yellow rectangles indicate locations of the magnified views shown on the right. **B** Quantification of the number of quaternary branches in a region 100 µm anterior to the PVD cell body. Statistical comparison was performed using Brown-Forsythe ANOVA with Dunnett’s multiple comparisons test (****, *p* < 0.0001; ***, *p* < 0.001; **, *p* < 0.01; *, *p* < 0.05) (*n* = 12, 16, 14, and 12 worms, respectively, in the order of presented genotypes). **C** Live animal imaging of developing PVD dendrites in L3 stage animals expressing a morphology marker. Cyan arrows indicate filopodia emerging from swelling in wild-type rescue. Yellow asterisks indicate swellings, including persistent tip swellings in UNC-34ΔEVH rescue animals, reminiscent of *unc-34 null* worms. Scale bar is 5 µm. **D** and **E** Quantification of filopodia and swelling formation as described in Fig. 2. Statistical comparison was performed with a Brown-Forsythe ANOVA with Dunnett’s multiple comparisons test (**, *p* < 0.01; *, *p* < 0.05; ns, *p* > 0.05; *n* = 11, 9, 19, and 11 worms, respectively, in the order of presented genotypes)

Having established that the ceWRC can bind to UNC-34 in vitro*,* we next asked whether the WRC-UNC-34 interaction is necessary for recruiting UNC-34 in PVD dendrites in vivo. To do so, we examined UNC-34 localization during PVD outgrowth by expressing an UNC-34:GFP fusion protein with a PVD-specific promoter in *unc-34* null animals (Fig. 5A). We found that, consistent with the importance of Ena/VASP proteins in elongating actin bundles, and with a recent report describing UNC-34 localization in PVD [24], UNC-34:GFP was enriched as puncta at the tips of growing dendrites (Fig. 5B, F; Additional file 6). Remarkably, UNC-34:GFP became enriched at the swelling sites before new branches started to emerge (Fig. 5D-F; Additional file 6). In contrast, when we expressed an UNC-34 lacking the EVH1 domain (UNC-34ΔEVH:GFP, Fig. 5A), the localization at both the tips of growing dendrites and the swelling sites before new branch initiation was significantly reduced (Fig. 5C-F; Additional file 7). Both wildtype UNC-34:GFP and UNC-34ΔEVH:GFP exhibit a decrease in signal after branch initiation (Fig. 5D) as a result of photobleaching during the imaging session. Note that removing the EVH1 domain from UNC-34 did not completely diminish its enrichment at the dendrite tips or swelling sites, which could be due other sequences in UNC-34, such as the EVH2 domain, binding the enriched F-actin [38, 39]. This interaction could account for the slight increase in UNC-34ΔEVH:GFP signal at 1 min before branch initiation (Fig. 5D). Nevertheless, the reduced enrichment of UNC-34ΔEVH:GFP at the swelling sites suggests the WRC-UNC-34 interaction plays an important role in recruiting UNC-34 to initiate filopodia formation. Finally, using the same constructs expressed in *unc-34* null animals with a PVD morphology marker, we asked whether the WRC-UNC-34 interaction was required for the ability of UNC-34 to promote PVD dendrite growth. We found that while the wild-type UNC-34:GFP construct significantly rescued quaternary branch formation, UNC-34ΔEVH:GFP showed no rescuing activity (Fig. 6A-B). Using live animal imaging, we found that the wild-type UNC-34:GFP rescue increased the occurrence of filopodia per dendrite, while UNC-34ΔEVH:GFP failed to do so (Fig. 6C-D; Additional files 8 and 9). In comparison, formation of swellings remained unchanged in all above *unc-34* null and rescued animals (Fig. 6E). Taken together, the above data support a model in which the WRC recruits UNC-34 to the swellings through the PPR-EVH1 interactions, and UNC-34 in turn promotes filopodia formation to initiate dendrite branching.

## Discussion

**Fig. 7:**
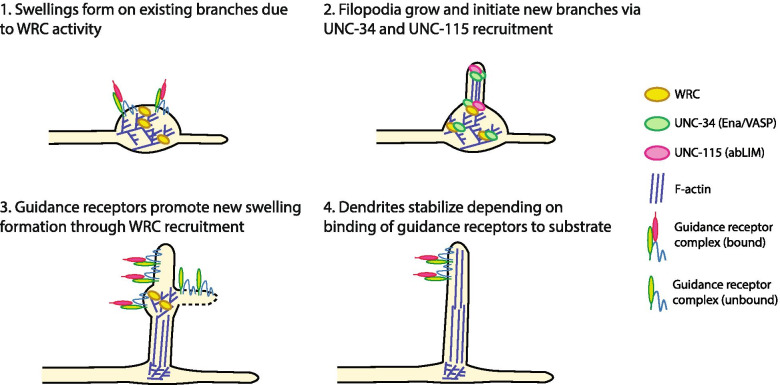
A two-step model for PVD dendrite development. 1) Guidance receptor complexes first recruit and activate the WRC, which in turn produces a branched actin network resulting in the formation of swellings. 2) The WRC (likely together with the branched actin network) recruits UNC-34 to the swellings through PRR-EVH1 interactions, which then, together with UNC-115, produce linear actin bundles to initiate filopodia outgrowth. UNC-34 continues to elongate actin filaments at the tips of filopodia. 3) As the new branch elongates, guidance receptor complexes can continue to recruit the WRC to the growth tip, causing pauses, new swellings, and new filopodia. 4) Filopodia are finally stabilized as new dendrites when guidance receptors bind to extracellular ligands, or retracted when ligands are not present

Our data explain how two distinct actin regulators, the WRC and Ena/VASP, coordinate their actin polymerization activity to drive dendritic branch formation (Fig. 7). In our model, the PVD guidance receptor complex first recruits the WRC through the cytosolic domains of the receptors DMA-1 and HPO-30 [23]. Activation of the WRC creates a branched actin network and morphological swellings akin to lamellipodia of a growth cone. The WRC further recruits UNC-34 to the swellings through direct PPR-EVH1 interactions. The branched actin filaments at swellings can then be bundled and extended by UNC-115/abLIM and UNC-34/Ena/VASP, creating filopodial structures to initiate and extend dendritic branches. The rapid extension of filopodia is likely mediated by the potent actin polymerization activity of UNC-34/Ena/VASP [12]. Once quaternary branches begin extending, additional interactions between external cues and the PVD guidance receptors stabilize the newly formed dendrite and further recruit the WRC, creating an iterative process to build and establish a dendritic arbor.

The observed pauses during branch extension are likely due to the WRC being recruited and activated by the guidance receptors DMA-1 and HPO-30. In addition to physical recruitment by these guidance receptors, WRC activation likely depends on additional regulation within the PVD dendrite, such as local Rac activation. Notably, prior work has established that TIAM-1/Tiam1, a Rac GEF, is recruited by the PVD guidance receptor DMA-1 [23, 25]. While the GEF activity of TIAM-1 does not seem to be necessary for its function, it is possible that TIAM-1 recruitment to the guidance receptor complex may help concentrate Rac near the WRC in order to promote WRC activation. Finally, while single mutants in other known WRC regulators such as Abl and Arf-6 [40, 41] have shown no obvious PVD phenotypes (data not shown), it remains possible that WRC activation can be regulated by other pathways that have yet to be identified during PVD dendrite morphogenesis. Following activation, the WRC likely subsequently recruits UNC-34/Ena/VASP to prime the actin networks for the next phase of dendrite extension.

### Diverse roles for Ena/VASP in dendrite morphogenesis

Recently, UNC-34 has also been reported to function in PVD dendrite morphogenesis by mediating self-avoidance between sister dendrites, functioning downstream of UNC-6/Netrin [24]. Here, our observations that *unc-34* mutants have fewer quaternary branches and greatly reduced filopodia formation during outgrowth suggest that UNC-34 acts cell-autonomously within PVD to promote dendrite outgrowth as well. We note that *unc-34* mutants often had primary dendrites that deviated from their normal trajectory to approach or merge with the sub-lateral line on which tertiary dendrites normally grow (Fig. 6A). This “wandering primary” phenotype was not consistently rescued by expressing UNC-34 in PVD, suggesting that UNC-34 might function in other cells, such as the ALA neuron [42, 43], to guide PVD primary dendrite development. Together, these observations indicate that Ena/VASP function is necessary for several aspects of neurite morphogenesis, including outgrowth, guidance, and self-avoidance.

### Swellings preconfigure dendrite branch sites

The morphology and dynamics of our observed “swellings” resemble those of “actin blobs” described by Nithianandam and Chien [44], which were actin-rich structures found at dendrite branch emergence sites in Drosophila class IV da neurons. Like actin blobs, swellings in PVD were observed both along dendrite shafts to initiate nascent branches and at the tips of outgrowing dendrites to promote further growth. These similarities suggest that different types of neurons in animals may share a common general mechanism to initiate dendrite branching, although different systems may vary in exactly using what molecules to implement this mechanism (for example, WRC-Arp2/3 [17], cofilin [44], and Cobl [45] have all been found to be major players in creating new sites of branch formation). Based on this general mechanism, formation of enlarged dendrite loci that are rich in branched actin networks preconfigures dendrite branching sites. Branched actin filaments in the local actin-rich structures can then be converted into actin bundles to promote dynamic outgrowth of filopodia, which is reminiscent of the “convergent elongation model” describing filopodia formation [35] and our proposed model of filopodia protrusion from swelling sites in PVD.

## Conclusions

In this study, we identified a series of cellular and molecular events that mediate the formation of a complex, branched dendritic arbor. We propose a mechanism by which the WRC establishes local actin-rich dendritic swellings and directly recruits UNC-34/Ena/VASP. Subsequently, UNC-34 builds filopodia and promotes rapid dendrite branching and outgrowth. Our findings are consistent with observations from *Drosophila* dendrite development, and suggest that the extension of linear, bundled actin from localized branched actin networks may be a general mechanism for initiating dendritic branches across different neuronal systems.

## Methods

### C. elegans strains and maintenance

C. elegans animals were raised on nematode growth medium (NGM) plates using OP50 Escherichia coli as a food source and maintained according to standard procedure unless otherwise noted [46]. Worms were raised at 20ºC and N2 Bristol worms were used as the wild-type strain. Data for quantification of mature dendrite morphology (e.g., number of quaternary branches) were taken at the L4 stage. For dynamic imaging of developing dendrites, synchronized worms were grown from bleached eggs until L3 stage.

### DNA manipulations and transgenes

Expression vectors were generated in the pSMdelta vector backbone (a derivative of pPD49.26).Transgenic *C. elegans* were prepared by microinjection into the gonad [47] using *Pmyo2* > *mCherry* (2 ng/ µl) as a co-injection marker. C. elegans strains used in this study are listed in Table 1.

**Table 1:** Worm strains used in the study

**Strain Name**	**Genotype and description**
TV15911	*wyIs592 [ser2prom3* > *myr-gfp* + *Podr-1* > *rfp]*
TV24718	*unc-34(gm104); wyIs592*
TV17281	*unc-115(ky275); wyIs592*
TV17232	*gex-3(zu196) / nT1; wyIs592*
TV23509	*gex-2(ok1603)/dpy-9(e12); wyIs592*
TV17113	*wve-1(ok3308)/ hT2; wdIs51 [F49H12.4 promoter* > *gfp* + *unc-119(* +*)]*
TV24999	*wyEx9925 [ser2prom3* > *unc-34:gfp* + *Pmyo2* > *mCherry]; wyIs581 [ser2prom3* > *myr-mCherry* + *Podr-1* > *gfp]*
TV26671	*wyEx10113 (ser2prom3* > *UNC-34ΔEVH:GFP* + *Pmyo2* > *mCherry); unc-34(gm104); wyIs581*
TV24987	*wyEx9925; unc-34(gm104); wyIs592*
TV25516	*wyEx10113 [ser2prom3* > *unc-34ΔEVH:gfp* + *Pmyo2* > *mCherry]; unc-34 (gm104); wyIs592*

### Confocal imaging of C. elegans

All images were acquired at room temperature in live *C. elegans.* For images of mature dendrite morphology, L4 stage animals were anesthesized using 10 mM levamisole in M9 buffer and mounted on 3% agarose pads. Worms were then imaged on a 3i spinning disk microscope with a CSU-W1 spinning disk (Yokogawa) using a C-Apochromat 40x / 0.9 NA water immersion objective. Images were acquired as z-stacks (0.75 µm/step, 15–18 steps) to cover the entire PVD dendritic arbor, and maximum-intensity projections were used for subsequent analyses.

For dynamic imaging of developing dendrite morphology, L3 worms were mounted onto a glass-bottom imaging dish (MatTek) as follows: Worms were picked using an eyebrow pick into a droplet of 5 mM levamisole on the glass bottom, and then covered with a 3% agarose pad. Worms were then imaged on a spinning disk microscope with a CSU-X1 spinning disk (Yokogawa) using a 40 × objective. Images were acquired every 90 s as z-stacks (0.75 µm/step, 12 steps) to cover the entire PVD dendritic arbor, and maximum-intensity projections were used for subsequent analyses.

For dynamic imaging of UNC-34:GFP, L3 worms were mounted onto a glass-bottom imaging dish as described above. Worms were imaged on a spinning disk microscope with a CSU-X1 spinning disk (Yokogawa) using a 63x/1.4NA objective. Images were acquired every 15 s as z-stacks (0.75 µm/step, 3 steps) to image a developing menorah, and maximum-intensity projections were used for subsequent analyses in Fiji.

### Quantification of dynamic imaging of dendrite morphology

Quantification of morphological features was done as follows: Filopodia formation was measured by manually counting the number of filopodia formed within a 12-min timeframe and normalized to the number of existing dendrites at the beginning of the timeframe. Swelling formation was measured by manually counting the number of swellings present at the end of the same 12-min timeframe and normalized to the number of existing dendrites at the end of the frame.

Filopodia were visually identified as protrusions no wider than 0.54 µm that extended at least 0.8 µm between 1.5-min frames. Swellings along dendrite shafts were visually identified as local increases in branch width of at least 30%, and occupying 0.54—1.4 µm of the shaft. Swellings at dendrite tips were allowed to be larger than 1.4 µm to accommodate the expanded tip swellings in *unc-34* mutants. Swellings were usually accompanied by a local increase in morphology marker intensity.

Quantification of growth speed was done as follows: Length of individual dendrites was tracked for at least 13 min at 1.5-min time intervals. Positive increases in length between frames were identified as positive growth events for quantification. The standard deviation (SD) of such positive growth events was measured for each dendrite, and is presented in Figs. 2 and 3 as “Variation in growth speed”. Lower SD indicates more consistent, gradual increases in dendrite length associated with lack of filopodia. The average of such positive growth events was also measured for each dendrite and is presented as “Average growth speed”.

### Quantification of UNC-34:GFP localization

UNC-34:GFP enrichment to swellings before branch initiation was measured as follows: GFP intensity was measured every 15 s at the location of a future branch initiation event starting 1.75 min before growth, and normalized to the average starting intensity across all measured dendrites. Statistical comparison was done between the -1.75 min (baseline) and -1.0 min timepoints.

UNC-34:GFP enrichment to dendrite tips was measured as % increase in GFP intensity at the tip of a growing dendrite as compared to GFP intensity along the shaft of the dendrite. Values from 4–10 branches were averaged per worm, and average values from a single worm were used for statistical analysis.

### Statistical analysis

All data are displayed as the mean ± standard error of the mean (SEM). Statistical comparisons were conducted using two-tailed Mann–Whitney tests (to test for differences between two groups) or Brown-Forsythe ANOVA with Dunnett’s multiple comparisons test (to test for differences between three or more groups). Sample sizes are indicated for each figure. ****, *p* < 0.0001; ***, *p* < 0.001; **, *p* < 0.01; *, *p* < 0.05 in all graphs. All statistical analyses and graph construction were performed using Prism 8.0 software (GraphPad Software, Inc).

### Protein purification and GST pulldown

GST-UNC-34 EVH1, MBP-WVE, MBP-Abi, and MBP-HSPC300 were expressed in Arctic Express (DE3) RIL (Agilent) or BL21 (DE3) T1^R^ (Sigma) cells after induction with 0.5 mM IPTG at 10 °C or 18 °C for 16 h. GST-UNC-34 EVH1 was purified through Glutathione Sepharose beads (GE Healthcare), followed by anion exchange chromatography using a Source 15Q column (GE Healthcare) at pH 7.0. MBP-WVE and MBP-Abi proteins were purified through amylose beads (New England Biolab). Trimeric subcomplexes containing WVE, Abi, and HSPC300 were assembled and purified by following previously described protocols [12, 23]. Briefly, individually purified subunits were mixed at equal molar ratio in the presence of 1% (w/v) NP40 and incubated on ice for 48 h. The assembled trimer was then purified by anion exchange chromatography through a Source 15Q column at pH 8.0, cation exchange chromatography by a Source 15S column at pH 6.0, and size exclusion chromatography through a Superdex 200 column (GE Healthcare).

GST pull-down assays were performed as previously described [23]. Briefly, 20 µL of GSH-Sepharose beads were mixed with 200 pmol of bait protein, 200 to 2,000 pmol of prey protein in 1 mL of pull-down buffer (50 or 100 mM NaCl, 10 mM HEPES pH 7.0, 5% (w/v) glycerol, 5 mM 2-mercaptoethanol, and 0.05% Triton X-100). The samples were mixed at 4 °C for 30 min, washed three times with each time using 1 mL of pull-down buffer, and eluted with 40 µL of elution buffer containing 30 mM reduced glutathione and 100 mM Tris pH 8.5. The eluant was examined by SDS-PAGE and Coomassie blue staining.

## Supplementary Information

**Additional file 1.** Example branch time-lapse imaging of wild-type dendrite growth using a myr-GFP morphology marker. Images are taken every 90 seconds. Arrowhead tracks tip of an individual dendrite during outgrowth; asterisks indicate “swellings” along the dendrite. Scale bar is 5 µm.

**Additional file 2.** Example branch time-lapse imaging of *unc-34(null)* dendrite growth using a myr-GFP morphology marker. Images are taken every 90 seconds. Yellow asterisks indicate persistent swellings that fail to generate filopodia and resemble lamellipodia-like growth cones. Scale bar is 5 µm.

**Additional file 3.** Example branch time-lapse imaging of *unc-115(null)* dendrite growth using a myr-GFP morphology marker. Images are taken every 90 seconds. Yellow asterisks indicate persistent swellings that fail to generate filopodia. Scale bar is 5 µm.

**Additional file 4.** Whole worm time-lapse imaging of wild-type dendrite growth using a myr-GFP morphology marker. Images are taken every 90 seconds. White arrowheads highlight three of many regions exhibiting quaternary dendrite growth. Magenta arrowhead highlights a secondary dendrite showing “rapid growth and pause” mode of growth, including swelling formation during pauses. Scale bar is 10 µm.

**Additional file 5.** Whole worm time-lapse imaging of *gex-3(null) *dendrite growth using a myr-GFP morphology marker. Images are taken every 90 seconds. Arrowheads point out dendrite growth events, although overall the dendritic arbor is much more static. Scale bar is 10 µm.

**Additional file 6.** Example branch time-lapse imaging of UNC-34:GFP localization using a myr-mCherry morphology marker. Images are taken every 15 seconds. White arrows indicate enrichment of UNC-34:GFP at tips of growing quaternary dendrites. White arrowheads indicate enrichment of UNC-34:GFP at branching sites prior to new branch initiation. Scale bar is 5 µm.

**Additional file 7.** Example branch time-lapse imaging of UNC-34ΔEVH:GFP localization using a myr-mCherry morphology marker. Images are taken every 15 seconds. White asterisk highlights outgrowth of a quaternary dendrite with large, lamellipodia-like growth cone that lacks UNC-34ΔEVH:GFP enrichment. Scale bar is 5 µm.

**Additional file 8.** Example branch time-lapse imaging of wild-type rescue experiment: UNC-34:GFP is expressed in an *unc-34(null)* worm. Dendrite growth is imaged using a myr-GFP morphology marker. Images are taken every 90 seconds. Yellow arrowheads indicate several filopodia events that emerge from swellings. Scale bar is 5 µm.

**Additional file 9.** Example branch time-lapse imaging of UNC-34ΔEVH rescue experiment:  UNC-34ΔEVH:GFP is expressed in an *unc-34(null) *worm. Dendrite growth is imaged using a myr-GFP morphology marker. Images are taken every 90 seconds. Yellow asterisks indicate persistent swellings that fail to generate filopodia and resemble lamellipodia-like growth cones. Scale bar is 5 µm.

**Additional file 10: Figure S1.** Formation of swellings during dendrite outgrowth in mutants lacking filopodia. As in Fig. 1B, the presence of a swelling at the tip of the dendrite is denoted with blue bars. Representative examples are provided for wildtype (A), *unc-34 *mutant (B), and *unc-115 *mutant (C) dendrites.

## Data Availability

All reagents and datasets used and/or analyzed during the current study are available from the corresponding author upon reasonable request.

## Abbreviations

Abi: Abelson interactor
abLIM: Actin-binding LIM protein
Ce3mer: A reconstituted trimer consisting of purified Abi, Wve, and HSPC300
Cobl: Cordon-bleu
da: Dendritic arborization
DCC: Deleted in Colorectal Carcinoma
DSCAM: Down syndrome cell adhesion molecule
Ena/VASP: Enabled/vasodilator-stimulated phosphoprotein
EVH: Ena/VASP homology
FL: Full-length
GEF: Guanine nucleotide exchange factor
GFP: Green fluorescent protein
GST: Glutathione-S-transferase
HSPC300: Haematopoietic stem/progenitor cell protein 300
PPR: Polyproline region
WAVE (WVE): WASP-family verprolin-homologous protein
WRC: WAVE regulatory complex
