# Supplementary figures and images for: A two-step actin polymerization mechanism drives dendrite branching
Source: Neural Dev. 2021 Jul 19;16:3. doi: 10.1186/s13064-021-00154-0 (PMC8290545; doi:10.1186/s13064-021-00154-0)

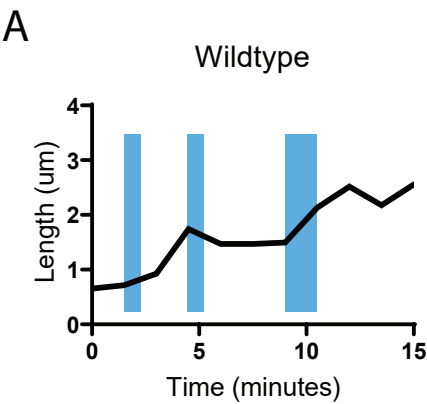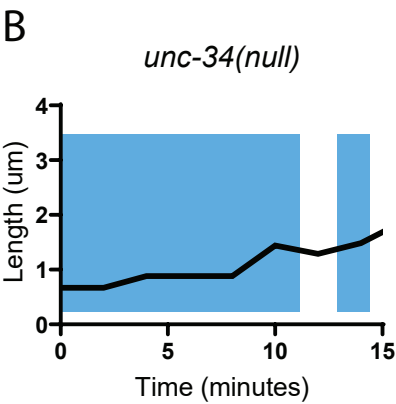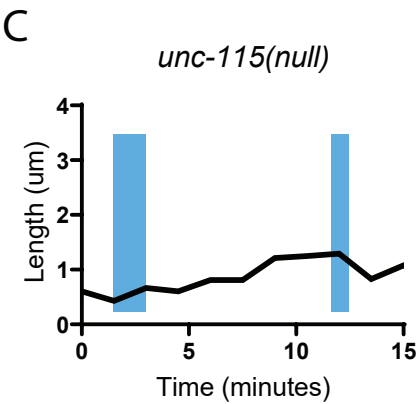

Supplement: Supplementary file 10 — Additional file 10: Figure S1. Formation of swellings during dendrite outgrowth in mutants lacking filopodia. As in Fig. 1B, the presence of a swelling at the tip of the dendrite is denoted with blue bars. Representative examples are provided for wildtype (A), unc-34 mutant (B), and unc-115 mutant (C) dendrites. [file 13064_2021_154_MOESM10_ESM.pdf]
